# Attitudes of Individuals with Gaucher Disease toward Substrate Reduction Therapies

**DOI:** 10.1007/s10897-017-0137-0

**Published:** 2017-08-13

**Authors:** Victoria F. Wagner, Hope Northrup, S. Shahrukh Hashmi, Joanne M. Nguyen, Mary Kay Koenig, Jessica M. Davis

**Affiliations:** 1 Genetic Counseling Program, The University of Texas MD Anderson Cancer Center UTHealth Graduate School of Biomedical Sciences, Houston, TX USA; 2 Department of Pediatrics, McGovern Medical School at The University of Texas Health Science Center, Houston, TX USA

**Keywords:** Gaucher disease, Type 1 Gaucher disease, Patient preferences, Therapy preferences, Substrate reduction therapy, Enzyme replacement therapy, Genetic counseling

## Abstract

Type 1 Gaucher disease (GD) is the most common lysosomal storage disorder. Previously, treatment for GD was limited to intravenous enzyme replacement therapies (ERTs). More recently, oral substrate reduction therapies (SRTs) were approved for treatment of GD. Although both therapies alleviate disease symptoms, attitudes toward SRTs and patient perceptions of health while using SRT have not been well established. Electronic surveys were administered to adults with GD and asked about treatment history, attitudes toward SRTs, and perception of health while using SRTs as compared to ERTs, if applicable to the participant. ERT users that were offered treatment with SRTs cited potential side effects, wanting more research on SRTs, and satisfaction with their current treatment regimen as reasons for declining SRTs. SRT users expressed convenience and less invasiveness as reasons for choosing SRTs. Additionally, those using SRTs most often perceived their health to be similar to when they previously used ERT. Participant responses illustrate that attitudes toward SRTs can be variable and that one particular treatment may not be ideal for all patients with GD depending on individual perceptions of factors such as convenience, invasiveness, or side effects. Thus, individuals with GD should be counseled adequately by healthcare providers about both ERTs and SRTs for treatment of GD now that SRTs are clinically available.

## Introduction

Gaucher disease is the most commonly inherited lysosomal storage disease and involves a deficiency of glucocerebrosidase (Meikle et al. 1999; Zhao and Grabowski 2002). As a result, the ability of lysosomes to break down endogenous glucosylceramide is impeded, leading to its accumulation within the cells of various organs (Brady et al. 1965; Esplin 1994). Type 1 Gaucher disease (hereafter abbreviated as GD) is considered non-neuropathic, accounts for 90% of all cases of Gaucher disease, and has increased prevalence in Ashkenazi Jewish populations (Beutler 1991; Esplin 1994). Progressive symptoms of GD include hepatosplenomegaly, anemia, thrombocytopenia, bone pain or fracture, and fatigue (Beutler 1991). While there is no cure for GD, enzyme replacement therapies (ERTs) were approved by the United States Food and Drug Administration (FDA) in 1994 for the treatment of GD symptoms (Esplin 1994). ERTs are administered via biweekly intravenous infusions and often require special facilities or staff for delivery due to potential for hypersensitivity reactions. As of 2014, the FDA and European Medicines Agency consider ERT to be the first-line treatment for GD (European Medicines Agency 2014).

Despite the success of many individuals managing GD with ERTs, some patients are unable to tolerate ERT or may experience adverse reactions (Van Rossum and Holsopple 2016). In 2003, the FDA approved a novel substrate reduction therapy (SRT), miglustat, for adults with GD and approved a second SRT, eliglustat, in 2014 (Cox et al. 2000; Poole 2014). SRTs are oral therapies and are non-inferior to ERTs with regard to several clinical measures and health-related quality of life instruments (Cox et al. 2015; Elstein et al. 2007; Lukina et al. 2014; Mistry et al. 2015; Pastores et al. 2005). Miglustat is taken three times per day and adjusted for renal creatinine clearance. Eliglustat is dosed according to *CYP2D6* genotype and taken once or twice daily depending on metabolizer status with adjustments for patients taking other CYP2D6 inhibitors. Eliglustat and miglustat have well established, but differing, side effect profiles (Cox et al. 2015; Elstein et al. 2007). Despite the possibility of adverse events, SRTs are reasonable alternatives for individuals with GD that develop adverse reactions to ERTs, cannot tolerate treatment with ERTs, or find intravenous ERT otherwise burdensome (European Medicines Agency 2014; Van Rossum and Holsopple 2016).

The availability of SRTs for patients with GD is the first example of an oral therapy utilized to treat a lysosomal storage disease traditionally managed with intravenous enzyme replacement. Additionally, emergence of SRTs represents the first time patients with GD have been able to exercise considerable choice regarding the type and delivery method of their therapy. As such, little is known about how individuals with GD view SRTs as a treatment option or what benefits or disadvantages patients may perceive surrounding these therapies. We sought to capture reasons why individuals with GD chose or declined use of SRTs, in order to add to existing knowledge about available treatment options for GD.

Furthermore, while efficacy of treating symptoms and disease progression is of great importance when evaluating a potential therapy, how a therapy affects an individual’s overall perception of their health and quality of life is also crucial to investigate. Because SRTs are oral therapies, they may impact patients differently, while providing similar symptom relief or management of GD. Therefore, patients with GD using SRTs may identify aspects of their health and quality of life that differ from when using ERTs in the past. Understanding potential differences in perceived patient quality of life, as well as the various reasons individuals may prefer or refuse use of SRTs for GD, is important for healthcare providers so they can appropriately care for and counsel patients with GD about the benefits and limitations associated with various treatment options. In order to accomplish the goals of the current study, we analyzed survey responses of individuals with GD that use either treatment method (ERTs or SRTs) regarding their views of SRTs and any differences in perceptions of health between currently using SRTs and when using ERTs in the past.

## Materials and Methods

### Participants

Invitations to participate in study questionnaires were posted in online forums consisting of patients with GD and their family members, including the Yahoo! Gaucher Disease group (https://groups.yahoo.com/neo/groups/gaucherdisease/info), the National Gaucher Foundation (NGF) website (http://www.gaucherdisease.org/), and the National Gaucher Foundation of Canada listserv (http://www.ngfcanada.com). Fliers advertising the study were also sent to physicians at GD treatment centers, as well as to the Metabolic Special Interest Group within the National Society of Genetic Counselors. Electronic advertisements were sent every three weeks during a collection period from October 12th, 2015 to January 7th, 2016. English speaking individuals 18 years of age or older with a self-reported diagnosis of GD met inclusion criteria for the study. Participants were informed during consent that two dollars would be donated to the NGF for each complete survey submitted. The total number of individuals with GD that the survey reached is unknown due to a paucity of data on the number of patients approached by the healthcare providers and the number of members in online support and information forums; therefore, a response rate could not be determined. Research data were collected through REDCap™, a secure electronic survey portal. The study received institutional review board exemption from the University of Texas Health Science Center (IRB #HSC-MS-15-0388)**.**

### Instrumentation

The demographic portion of the study questionnaire was comprised of questions regarding age, sex, ethnicity and ancestry, country of residence, education, and marital status. Other sections of the survey included questions pertaining to history of GD, other medical history, and treatment history for GD. Participants answered two open-ended questions about their attitudes toward SRTs if they had been offered them in the past. Individuals using SRTs to treat GD that used ERTs in the past were asked five additional forced choice questions about their perceptions of their health in five different categories (general health, physical ability, emotional health, social interactions, and satisfaction with life) across the use of the two treatment options. No survey items were deemed mandatory.

### Data Analysis

Survey responses were recorded and forced choice question answers were entered into STATA®, a statistical software program for data analysis (v. 13. StataCorp LP, College Station, TX). Frequencies with percentages and medians with interquartile ranges (IQRs) were calculated for all categorical and non-normally distributed continuous data, respectively. A Mann-Whitney test was used to compare data between groups and statistical significance was assumed at a Type I error rate of 5%. Descriptive statistics were used for the remaining components of the questionnaire. Participant answers to open-ended questions were analyzed using conventional content analysis and grouped by content to compare the presence of themes across treatment populations (Hsieh and Shannon 2005). Two authors (Wagner VF and Davis JM) independently examined participant answers to identify these themes and differences were discussed before reaching agreement on how to code responses. Due to the open-ended nature of the response, more than one theme was occasionally applicable to an individual’s answer.

## Results

### Sample Description and Demographics

**Fig. 1:**
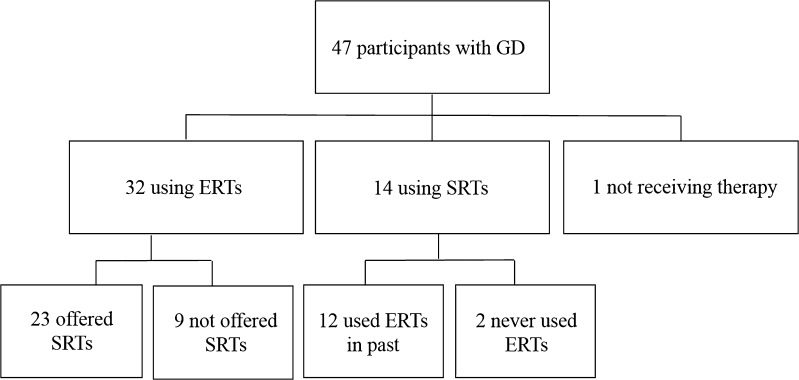
Flow diagram of participant treatment history for type 1 Gaucher disease

Forty-seven adults with GD completed the study questionnaire. Thirty-two of these participants reported using ERTs (68%), while 14 reported using SRTs (30%), and one individual reported no current therapy for GD at the time of the study (2%). Twenty-three out of 32 ERT users (72%) had been offered a SRT by a physician in the past. Twelve of the 14 current SRT users (86%) also had a past history of ERT use for treatment of GD (Fig. 1). Responses by the 14 current SRT users and 23 current ERT users that were offered SRTs by physicians were the only ones analyzed for the purpose of this study (*n* = 37). Participants did not further specify which form of ERT or SRT they utilize for treatment.

Current median age of participants was 51 years (IQR: 32–59; range: 18–78). The median age that participants were diagnosed with GD was 23 years (IQR: 5–40; range: 1–59). Current age was not significantly different between participants who switched from ERT to SRT and those who decided to stay on ERT when offered SRT (*p* = 0.365). In terms of disease treatment, the median age that individuals began therapy for GD was 35 years (IQR: 18–50; range: 1–62). Demographic and background information of participants are presented in Table 1, including age, sex, ethnicity, Jewish ancestry, country of residence, education level, marital status, and length of time receiving treatment.

**Table 1:** Demographic information and treatment histories of participants with Gaucher Disease (*N* = 37)

	*n*	Percentage^a^
Age		
18–40	13	35
41–60	16	43
61–78	8	22
Sex		
Female	27	73
Male	10	27
Ethnicity		
Caucasian	36	97
Hispanic/Latino	1	3
Jewish ancestry		
Yes	24	65
No	11	30
Not sure	2	5
Country of residence		
United States	32	87
Other	5	13
Education		
College degree or higher	26	70
Some college or less	11	30
Marital status		
Single, divorced, or widowed	20	54
Married	17	46
Length of time on ERT (*n* = 23, 62%)		
Less than 1 year	0	0
Between 1 and 5 years	4	18
More than 5 years	18	78
No response	1	4
Length of time on SRT (*n* = 14, 38%)		
Less than 1 year	7	50
Between 1 and 5 years	5	36
More than 5 years	1	7
No response	1	7

^a^Percentage refers to the percentage of total participants reporting a demographic or characteristic in each of the nine separate categories

Participants were asked to indicate any symptoms of GD they were experiencing around the time of survey administration including enlarged liver, enlarged spleen, anemia, bone pain, fatigue, frequent bruising, bleeding problems, enlarged abdomen, fractures, liver problems, or other symptoms. Disease variability was present, with 24% reporting no current symptoms (*n* = 9), 52% reporting 1–4 symptoms (*n* = 19), 24% reporting 5–8 symptoms (*n* = 9), and no participants reporting greater than 8 symptoms of GD. The most common symptom was fatigue (*n* = 24, 65%). Additionally, individuals were asked to report medical histories of any other chronic conditions including back problems, cancer, hypertension, diabetes, arthritis, chronic allergies, vision problems, sciatica, hearing problems, chronic lung disease, and other chronic conditions. The most common comorbidity reported was hypertension (*n* = 10, 27%). Thirty-eight percent of individuals cited no chronic conditions other than GD (*n* = 14), 52% indicated that they had 1–3 additional chronic conditions (*n* = 19), 10% reported 4–6 other chronic conditions (*n* = 4), and no one experienced greater than 6 additional chronic conditions (Table 2).

**Table 2:** Clinical symptoms of Gaucher Disease (GD) and medical histories (*N* = 37)

	*n*	Percentage
Symptoms of GD^a^		
Fatigue	24	65
Bone pain	13	35
Enlarged liver	11	30
Enlarged spleen	10	27
Frequent Bruising	7	19
Anemia	6	16
Enlarged abdomen	6	16
Other symptom of GD	5	14
Fractures	4	11
Nose bleeds/bleeding problems	4	11
Liver problems	3	8
Medical History^a^		
Hypertension	10	27
Back problems	8	22
Arthritis	7	19
Other chronic condition	7	19
Chronic allergies	5	14
Hearing problems	3	8
Vision problems	3	8
Cancer	2	5
Diabetes	2	5
Sciatica	2	5
Chronic lung disease	0	0

^a^Symptoms and comorbidities related to medical history were not mutually exclusive options for survey respondents

### Attitudes toward SRTs

Individuals currently using ERT who had been offered SRTs by a physician (*n* = 23) were asked to provide the reasons that contributed to their decision not to use SRTs (Table 3). Free responses represented six distinct themes: potential or experienced side effects of SRTs (indicated by 16 individuals), not enough research on SRTs (*n* = 8), satisfaction with ERT (*n* = 5), convenience (*n* = 2), barriers to SRT use (*n* = 1), and cost or insurance coverage (*n* = 1). Fourteen current SRT users were asked to provide reasons that contributed to their decision to use SRTs (Table 4). Six main themes emerged: convenience of SRTs (*n* = 7), less invasive than ERTs (*n* = 4), reaction to ERT (*n* = 1), continued SRT after FDA study (*n* = 1), cost or insurance coverage (*n* = 1), and recommended by a doctor (*n* = 1).

**Table 3:** Reasons cited for declining SRT current ERT users (*N* = 23)

Reasons cited for declining SRT	Frequency	Selected quotes from participants
Side effects of SRTs	16	“Very active in the Gaucher community and I am aware of some of the side-effects … experienced.” 66 year-old female “The possibility [of] long-term effects.” 18 year-old male “I had trouble with a lot of acid reflux and heartburn with [an SRT] that I couldn’t get controlled….” 61 year-old female
Not enough research on SRTs	8	“I feel like [SRTs are] being pushed on us, which makes me uneasy…. The drug is too new, I will wait until there is more conclusive research before considering.” 32 year-old female “I am not convinced that SRT is an equal or more desirable treatment path than ERT.” 56 year-old male
Satisfaction with current therapy	5	“My physician offered [SRT] as something to think about, but I chose not to consider it because ERT works so well for me.” 29 year-old female “I am happy with my ERT….” 78 year-old female
Convenience	2	“[I] don’t want to miss a pill… [I] infuse myself so it’s convenient and then I don’t have to deal with it for 2 more weeks.” 49 year-old female
Barriers to SRT use	1	“[I] have not had time to do extra tests [for SRT].” 38 year-old female
Cost or insurance coverage	1	“[I am] on Medicare, so it is currently too expensive to use [SRTs] because it would fall under prescriptions instead of medical, as the infusions are.” 66 year-old female

**Table 4:** Reasons cited for using SRT by current SRT users (*N* = 14)

Reasons cited for using SRT	Frequency	Selected quotes from participants
Convenience	7	“It is very convenient to take [an SRT] because I can take pills instead of driving to a facility to take [an ERT].” 28 year-old female “Taking an oral [medication] is much easier than infusions.” 57 year-old female “I loved [ERT] but the amount of time it took for an infusion plus travel to hospital turned into an all day event.” 24 year-old female “[SRTs are] more convenient than infusions.” 51 year-old female
Less invasiveness of SRTs	4	“Lack of needles.” 48 year-old female “Less invasive than ERT; less interference with lifestyle.” 63 year-old male “[I] hate needles.” 44 year-old female
Continued after clinical trial	2	“I joined an SRT drug study during the ERT shortage and stayed on SRT after FDA approval.” 52 year-old female
Reaction to ERTs	1	“[I] developed a serious allergic reaction to [ERT]....” 32 year-old female
Cost or insurance coverage	1	“Billing was also a huge factor…. Very stressful.” 24 year-old female
Doctor recommended	1	“My doctor recommended [an SRT].” 51 year-old female

### Perceptions of Health in SRT Users

**Fig. 2:**
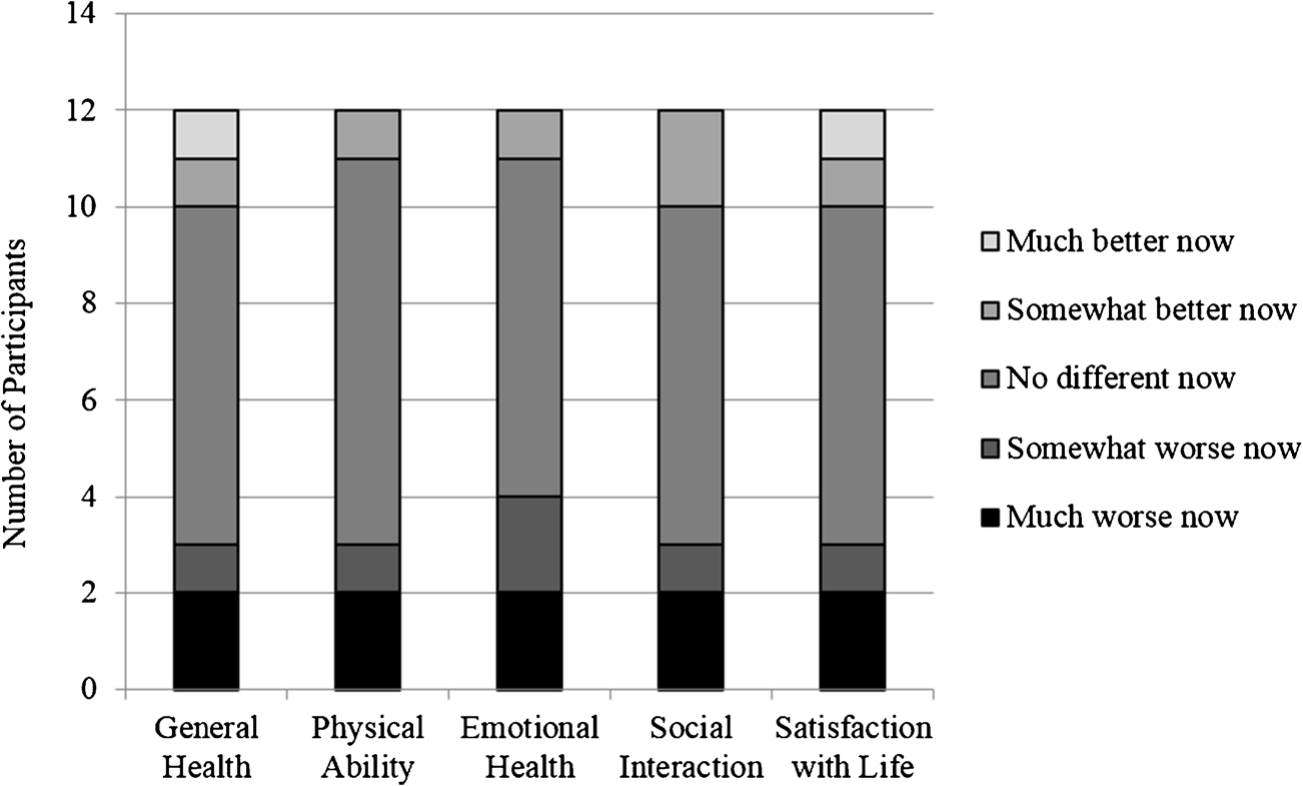
SRT users’ perceptions of current health as compared to health during ERT use

Twelve current SRT users that used ERTs in the past compared their perceptions of health while on each treatment with regard to five health categories: “general health,” ability to complete everyday activities or “physical ability,” “emotional health,” “social interactions,” and “satisfaction with life.” More than half of the subgroup reported no difference in their perception of health while using SRTs as compared to ERTs with regard to general health, physical ability, emotional health, social interactions, and satisfaction with life (Fig. 2). Between one and two current SRT users per category reported having a somewhat better or much better perception of health while using SRTs as compared to when using ERTs. The same two participants reported much worse health while using SRTs than when using ERTs for each of the five health categories. Both of these participants indicated in free responses that their reason for using SRTs was aversion to needles for infusion of ERTs.

## Discussion

Although ERTs are the traditional standard of treatment for GD, the introduction of SRTs for the treatment of GD has piqued the interest of physicians and patients alike regarding this new class of therapies. Clinical trials have established that SRTs are non-inferior to ERTs with regard to various symptoms and patient quality of life (Cox et al. 2015; Elstein et al. 2007; Lukina et al. 2014; Mistry et al. 2015; Pastores et al. 2005). While these data on SRT use by patients with GD are of great importance, individuals with GD have not been previously asked about their thoughts or experiences regarding the use of SRTs to treat or manage GD. The goal of this study was to add to the already existing information regarding the use of SRTs for treatment of GD with a particular focus on patient attitudes toward and perceptions of SRTs.

When asked about why current ERT users declined SRTs, the most common reasons cited were concern for side effects, feeling as though there is not enough research on SRTs, and satisfaction with their ERT. Previous research of therapy preferences in patients with hemophilia has shown that side effects are a major influence on patient preferences for therapy (Chaugule et al. 2015; Mohamed et al. 2011). Our findings show that the risk of side effects as a marker for therapy preference extends to patients with GD now that there are two types of drug classes available for treatment. The idea that individuals who were satisfied with ERT use and were comfortable with the routine of their bi-weekly infusions was unsurprising, since greater than three quarters of assessed ERT users had used ERTs for more than five years. Other investigations of patient preferences for treatment of chronic hematological conditions are consistent with this theme and showed that, even if new treatments become available, many patients feel uncertain about or refuse change in treatment, at times due to satisfaction with their current treatment regimen (Renzi et al. 2016; Trachtenberg et al. 2014).

Alternatively, the most common reasons current SRT users cited for using SRTs were convenience and their less invasive nature. Other SRT users mentioned continuing SRTs after clinical trials or because of a doctor’s recommendation. These findings mirror reasons why other patient populations, including those with multiple sclerosis, have switched to new forms of therapies (Salter et al. 2014). The themes of the open-ended responses of ERT users that declined use of SRTs and current SRT users represent important considerations not only for the potential introduction of additional therapies for GD in the future, but also for other storage disorders or inherited diseases with available treatment options. Furthermore, the variety of attitudes of individuals with GD toward SRTs is remarkable and illustrates that healthcare providers, including genetic counselors and other genetics providers, should take patient preferences into account when discussing treatment options for GD now that multiple forms of therapy are available.

Comparing patient perceptions of health across the two different types of therapy for GD also provided added insight into aspects of the patient experience concerning SRTs. SRT users that previously used ERT most often reported no difference in health when asked to compare aspects of their current health to their health while using ERT. The finding that the majority of current SRT users in this study perceived no difference in multiple aspects of their health across therapy types was unexpected. Of note, two respondents that considered their health to be “much worse now with SRT” across all categories than when they used ERT reported side effects with SRT use in their open-ended responses. Evidence suggests that use of ERTs and SRTs improves health-related quality of life (HRQoL) for untreated individuals with GD (Mistry et al. 2015). Additionally, no significant difference in HRQoL was found in individuals following a treatment switch from an ERT, imiglucerase, to an SRT, miglustat (Cox et al. 2015). However, no study to-date had investigated comparisons of health across both types of therapy for the same patient with GD. Our findings agree with prior research and reveal that, overall, patients with GD that use SRTs typically do not perceive their health to be different from when they used ERTs in the past for treatment of GD. Nonetheless, it may not be appropriate to extrapolate that observation to each individual with GD. While some patients with GD may find advantages of SRTs as a type of treatment for GD, as demonstrated by our cohort’s free responses, patients experiencing HRQoL-reducing side effects when using SRTs may perceive or experience worse health than when using ERTs.

### Limitations

A notable limitation of the study is the small sample size, particularly for those individuals that use SRTs. SRTs are only recently clinically available for patients with GD and, as our results indicate, patients already using ERTs may be resistant to a therapy switch. Despite few participants, many themes regarding patient attitudes towards therapy options had reproducibility within the sample. As such, it is likely that these factors influence treatment related decision-making in a larger population with GD. However, without a larger sample, it is possible that some of these themes may not represent global views of individuals with GD that are considering SRT use.

Many participants were recruited from electronic information and support communities related to GD. While our group did extend participation to patients of metabolic treatment centers with GD to avoid sample bias, it is possible that responses of individuals with GD that are active members of these electronic communities may not have responses representative of the average adult with GD (Gawlinski et al. 2013). Participants did not report their source of learning about this study, so a reliable response rate from varying sources is unavailable.

Additionally, side effect profiles of eliglustat and miglustat are well described in the available literature and suggest that side effects of eliglustat may be milder than for miglustat (Cox et al. 2015; Elstein et al. 2007; Lukina et al. 2014; Pastores et al. 2005). The type of SRT that individual participants used for treatment of GD was not recorded in this study and, therefore, we cannot assess the potential for difference in how each form of SRT may affect individuals’ perceptions of health. This is especially important to consider in light of some individuals reporting side effects while using SRTs. While some features of SRTs used for treatment of GD are similar across eliglustat and miglustat, such as the route of medication administration, it is important to recognize that these two medications are not equal in every regard.

### Research Recommendations

As previously stated, corroboration of these themes or identification of other themes regarding attitudes of patients with GD toward SRTs in larger numbers is desired. Over half of participants using SRTs had been using this form of therapy for less than 1 year and it will also be important to see if individuals’ perceptions of health are comparable after a longer period of treatment with SRT. Now that patient attitudes toward SRTs for treatment of GD have been identified, additional research on the type of guidance that individuals may find helpful from their genetics providers regarding therapy options would add to practical knowledge of how genetic counselors and physicians can most effectively address these treatment-specific needs and concerns, if present. Furthermore, it would be interesting to survey patients with other treatable metabolic conditions that utilize ERT to determine if attitudes toward oral therapies would be similar or different. Finally, if SRTs become approved for use in pediatric populations in the future, studies may look to see if similar perceptions of SRTs exist among those who have had the option of using SRTs for the majority of the time that they have received treatment for GD.

### Practice Implications

The outcomes of the current study indicate that patient attitudes toward SRTs can be extremely diverse and are an important factor that could impact personal preferences for treatment of GD. Though many view ERTs as the first-line therapy for GD and SRTs as an acceptable alternative, attitudes toward and perception of SRTs for treatment of GD are significant contributions to individual preferences for and decision-making regarding therapy regimens. Therefore, genetics providers should be aware of the differing attitudes that patients may have concerning treatment options for GD, including an assortment of perceived advantages and disadvantages of SRTs, and use this knowledge to counsel patients extensively when discussing therapy options. Specifically, genetic counselors and other providers should consider having open discussions with their patients about their prior knowledge of treatment for GD and their perceptions of different therapy options at the beginning of treatment and as other approved treatment options become available.
